# Understanding the phase separation characteristics of nucleocapsid protein provides a new therapeutic opportunity against SARS-CoV-2

**DOI:** 10.1007/s13238-021-00832-z

**Published:** 2021-03-26

**Authors:** Dan Zhao, Weifan Xu, Xiaofan Zhang, Xiaoting Wang, Yiyue Ge, Enming Yuan, Yuanpeng Xiong, Shenyang Wu, Shuya Li, Nian Wu, Tingzhong Tian, Xiaolong Feng, Hantao Shu, Peng Lang, Jingxin Li, Fengcai Zhu, Xiaokun Shen, Haitao Li, Pilong Li, Jianyang Zeng

**Affiliations:** Institute for Interdisciplinary Information Sciences, Tsinghua University, 100084, Beijing, China; Beijing Advanced Innovation Center for Structural Biology & Frontier Research Center for Biological Structure, School of Life Sciences, Tsinghua University, 100084, Beijing, China; Tsinghua-Peking Center for Life Sciences, Tsinghua University, 100084, Beijing, China; Institute for Interdisciplinary Information Sciences, Tsinghua University, 100084, Beijing, China; Silexon AI Technology Co., Ltd, 210033, Nanjing, China; NHC Key Laboratory of Enteric Pathogenic Microbiology, Jiangsu Provincial Center for Diseases Control and Prevention, 210009, Nanjing, China; Institute for Interdisciplinary Information Sciences, Tsinghua University, 100084, Beijing, China; Bioinformatics Division, BNRIST/Department of Computer Science and Technology, Tsinghua University, 100084, Beijing, China; Protein Preparation and Identification Facility, Technology Center for Protein Science, Tsinghua University, 100084, Beijing, China; Institute for Interdisciplinary Information Sciences, Tsinghua University, 100084, Beijing, China; Institute for Interdisciplinary Information Sciences, Tsinghua University, 100084, Beijing, China; Institute for Interdisciplinary Information Sciences, Tsinghua University, 100084, Beijing, China; Institute of Pathology, Tongji Hospital, Tongji Medical College, Huazhong University of Science and Technology, 430074, Wuhan, China; Institute for Interdisciplinary Information Sciences, Tsinghua University, 100084, Beijing, China; Institute for Interdisciplinary Information Sciences, Tsinghua University, 100084, Beijing, China; NHC Key Laboratory of Enteric Pathogenic Microbiology, Jiangsu Provincial Center for Diseases Control and Prevention, 210009, Nanjing, China; NHC Key Laboratory of Enteric Pathogenic Microbiology, Jiangsu Provincial Center for Diseases Control and Prevention, 210009, Nanjing, China; Center for Global Health, Nanjing Medical University, 210009, Nanjing, China; Convalife (Shanghai) Co., Ltd, 201203, Shanghai, China; Tsinghua-Peking Center for Life Sciences, Tsinghua University, 100084, Beijing, China; Ministry of Education Key Laboratory of Protein Sciences, Beijing Advanced Innovation Center for Structural Biology, Beijing Frontier Research Center for Biological Structure, Department of Basic Medical Sciences, School of Medicine, Tsinghua University, 100084, Beijing, China; Beijing Advanced Innovation Center for Structural Biology & Frontier Research Center for Biological Structure, School of Life Sciences, Tsinghua University, 100084, Beijing, China; Tsinghua-Peking Center for Life Sciences, Tsinghua University, 100084, Beijing, China; Institute for Interdisciplinary Information Sciences, Tsinghua University, 100084, Beijing, China

**Dear Editor**

To date, tens of millions of people have been infected with severe acute respiratory syndrome coronavirus 2 (SARS-CoV-2), causing the outbreak of the respiratory disease named the coronavirus disease 2019 (COVID-19). As a newly emerged member of the coronavirus family, SARS-CoV-2 is an enveloped positive-strand RNA virus, which has probably the largest genome (approximately 30 kb) among all RNA viruses. The nucleocapsid (N) protein of SARS-CoV-2 is mainly responsible for recognizing and wrapping viral RNA into helically symmetric structures (Malik, 2020). It was also reported that N protein can boost the efficiency of transcription and replication of viral RNA, implying its vital and multifunctional roles in the life cycle of coronavirus (Surjit and Lal, 2008; Chang et al., 2014). Recently, several independent research teams have reported that N protein of SARS-CoV-2 is capable of undergoing liquid-liquid phase separation (LLPS) (Iserman et al., 2020; Perdikari et al., 2020; Savastano et al., 2020).

Here, we comprehensively determined the characteristics of the phase separation driven by the N protein of SARS-CoV-2 (termed SARS-CoV-2 N), and found that LLPS is involved in the interplay between the N protein-viral RNA complex of SARS-CoV-2 (termed SARS-CoV-2 N-RNA) and other viral proteins, such as nsp12. Importantly, we identified two small molecules targeting the SARS-CoV-2 N protein, which can intervene the phase separation properties of the N protein-viral RNA-nsp12 (termed SARS-CoV-2 N-RNA-nsp12) complex, thus probably improving the accessibility of other antiviral drugs (e.g., remdesivir) to their viral targets (e.g., nsp12/RdRp).

First, IUPred2 (Erdos and Dosztanyi, 2020) and PLAAC (Lancaster et al., 2014) programs showed that SARS-CoV-2 N is highly disordered, and contains three intrinsically disordered regions (IDRs), with one also displaying prion-like activity (Figs. 1A and S1). Then, we expressed and purified the recombinant SARS-CoV-2 N protein with an mEGFP-tag (a monomeric variant of EGFP, A206K) or a His-tag using a prokaryotic expression system to understand the properties of N-driven LLPS *in vitro* (Fig. S2A and S2B). Confocal fluorescence microscopy showed that SARS-CoV-2 N was readily self-associated to form numerous micron-sized spherical condensates (Fig. 1B and 1C). Further time-lapse observations revealed that the SARS-CoV-2 N condensates fused and coalesced into larger ones upon their intersections (Fig. 1D and Video S1), verifying the liquid-like properties of SARS-CoV-2 N condensates. We also used fluorescence recovery after photobleaching (FRAP) to deeply study the dynamics of internal molecules within the N protein condensates. Recovery of fluorescence within the bleached regions (Fig. 1E) showed that SARS-CoV-2 N can partially freely diffuse within the condensed phase, consistent with their liquid-like behavior. In addition, phase condensation of SARS-CoV-2 N was sensitive to the increase of ionic strength, suggesting that electrostatic interactions are important for its condensation (Fig. S3). Consistently, we also demonstrated that SARS-CoV-2 N can also undergo LLPS *in cellulo* and display liquid-like behavior, by ectopically expressing an mCherry-tagged version of SARS-CoV-2 N in Vero E6 cells (Fig. 1F, 1G, 1H and Video S2).

**Figure 1:**
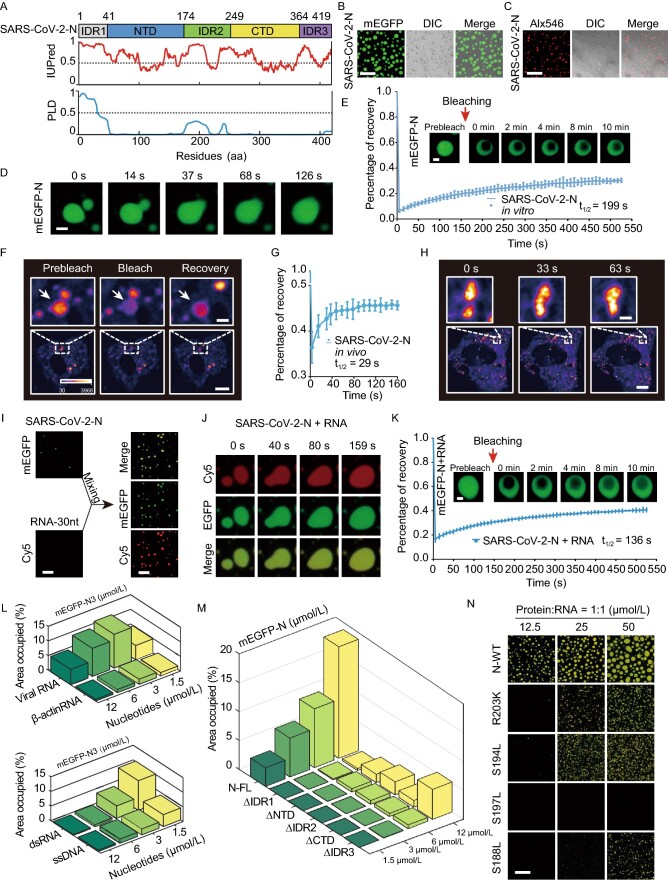
**SARS-CoV-2 N undergoes phase separation and viral RNA facilitates phase condensation of SARS-CoV-2 N**. (A) Bioinformatic analysis of the amino acid sequence of SARS-CoV-2 N. Schematic representation of the domain structure is shown on the top. IDR, intrinsically disorder region; NTD, N-terminal domain; CTD, C-terminal domain; IUPred, prediction of intrinsic disorder; PLD, prediction of prion-like region (PLAAC). (B) *In vitro* phase separation assays of 25 μmol/L mEGFP tagged SARS-CoV-2 N protein (mEGFP-N). Scale bar, 20 μm. (C) *In vitro* phase separation assays of 25 μmol/L full-length SARS-CoV-2 N protein labeled with Alx546. Scale bar, 20 μm. (D) Fusion of mEGFP-N (50 μmol/L) condensates. Data are representative of three independent experiments. Scale bar, 2.5 μm. (E) *In vitro* FRAP analysis of the condensates (*n* = 3) of mEGFP-N (3 µmol/L). Top, representative snapshots of condensates before and after bleaching. Bottom, average fluorescence recovery traces of mEGFP-N condensates. Data are representative of three independent experiments and presented as mean ± SD. Scale bar, 1 μm. (F) *In cellulo* FRAP analysis of the puncta of the expressed mCherry-N (SARS-CoV-2 N tagged with mCherry) in Vero E6 cells. Insets, representative snapshots of puncta before and after bleaching. The bleached punctum is marked with an arrow. Scale bars, 10 µm (bottom), 2 µm (insets). (G) The average fluorescence recovery traces of the puncta (*n* = 3) of the expressed mCherry-N protein in Vero E6 cells presented in (F). Data are representative of three independent experiments and presented as mean ± SD. (H) Fluorescence time-lapse microscopy of Vero E6 cells expressing mCherry-N. In (F) and (H), the “Red Fire” lookup table in the NIS-Elements Viewer was used to highlight the intensity difference. Two mCherry-N puncta are zoomed-in. Scale bars, 5 μm (bottom), 1 μm (insets). (I) Left, *in vitro* phase separation assays of mEGFP-tagged SARS-CoV-2 N (mEGFP-N) alone (375 nmol/L) or Cy5-labeled 30-nt viral RNA alone (1.5 μmol/L). Right, puncta formed by mEGFP-N (375 nmol/L) mixed with 30-nt viral RNA (375 nmol/L) *in vitro*. Scale bar, 5 µm. (J) Fusion upon contact of the condensates of mEGFP-N (50 μmol/L) with 30-nt viral RNA (25 μmol/L). Scale bar, 2.5 µm. (K) *In vitro* FRAP analysis of the condensates (*n* = 3) of mEGFP-N (3 µmol/L) with 30-nt viral RNA (1.5 µmol/L). Top, representative snapshots of condensates before and after bleaching. Bottom, average fluorescence recovery traces of mEGFP-N with viral RNA in condensates. Data are representative of three independent experiments and presented as mean ± SD. Scale bar, 1 μm. (L) Quantitative comparison of phase condensation of mEGFP-N with different nucleic acids. (M) Quantitative comparison of the phase condensation of full-length and truncated mEGFP-N proteins with 30-nt viral RNA (1.5 µmol/L). In (L) and (M), %Area Occupied = [Sum of area occupied by N protein condensates] × 100/[The whole area]. (N) *In vitro* phase separation assays of the Alx546-labeled wild-type (WT) N protein and four mutants with mutations on the serine-arginine (SR) rich region with viral RNA of different concentrations. The ratio of N protein to viral RNA was 1:1. Scale bar, 20 μm. The reaction buffer of *in vitro* phase separation assays in (B), (D and E) and (I–N) consists of 20 mmol/L HEPES, pH 7.4, 150 mmol/L NaCl, 5% glycerol, 1 mmol/L EGTA and 1 mmol/L MgCl_2_. The assays in (C) were performed in 20 mmol/L Tris-HCl, pH 7.5 and 150 mmol/L NaCl

Given that N protein of coronavirus prefers to bind to the intergenic regions and exhibits high binding affinity with the UCUAA pentanucleotide repeats (Stohlman et al., 1988; Nelson et al., 2000; Lin et al., 2014), we found that the addition of this viral RNA to the purified SARS-CoV-2 N solution at a physiological salt concentration (150 mmol/L NaCl) resulted in robust co-phase separation of these two components, which was more predominant than that of a single one alone (Fig. 1I). Compared to the condensates of SARS-CoV-2 N alone, the droplets of N protein-RNA (termed SARS-CoV-2 N-RNA) complex were more spherical (Fig. 1J and Video S3) and exhibited slightly higher molecular exchange rates than those of N protein alone (Figs. 1K and S4). Moreover, although the synthesized viral RNA, the host-derived RNA (β-actin RNA), the dsRNA and the ssDNA (both derived from viral RNA sequences) all exhibited similar behaviors in inducing phase separation, viral RNA displayed the most prominent effect (Figs. 1L and S5A) in regulating the LLPS of SARS-CoV-2 N.

To determine the contributions of individual domains of SARS-CoV-2 N to its phase separation, we designed and expressed five truncations (ΔIDR1, ΔNTD, ΔIDR2, ΔCTD, ΔIDR3) (Fig. S5B) using a prokaryotic expression system and revealed the remained LLPS ability in all truncations, though much weaker than that of the full-length (Figs. 1M and S5C). Besides, we analyzed all the missense mutations within the N protein region (between genome positions 28,274 and 29,530) according to the China National Center for Bioinformation, 2019 Novel Coronavirus Resource (https://bigd.big.ac.cn/ncov?lang=en, July 6th, 2020). Notably, the serine-arginine (SR) rich region within the IDR2 domain of SARS-CoV-2 N was the hot spot harboring 7 of the top 10 most frequent mutations (Fig. S6). Subsequently, we expressed and purified several mutant proteins *in vitro,* including R203K, S194L, S197L and S188L mutants (4 among the top 5 mutations) (Fig. S2B). Interestingly, these mutants displayed markedly weaker phase separation than the wild-type N protein (Fig. 1N), in agreement with other studies showing that the SR region impacts the multivalent RNA-protein or protein-protein interactions, and phosphorylation on the SR region could reduce these interactions (Carlson et al., 2020).

Our previous study has identified two poly ADP-ribose polymerase (PARP) inhibitors with antiviral activities, CVL218 and PJ34, as the potential binding small molecules of SARS-CoV-2 N (Ge et al., 2020). Therefore, we wondered whether these small molecules can impair SARS-CoV-2 infection by modulating the phase separation properties of N protein. Surface plasmon resonance (SPR) analyses confirmed their binding affinity with SARS-CoV-2 N, with CVL218 showing a higher binding affinity (*K*_D_ = 4.7 μmol/L, Fig. 2A) than PJ34 (*K*_D_ = 696 μmol/L, Fig. 2B). Next, we observed that the addition of CVL218 or PJ34 (20 μmol/L) led to larger sizes and faster recovery rates of the fluorescence intensity of the SARS-CoV-2 N-RNA condensates than the DMSO group *in vitro* (Fig. 2C and 2G–J). Moreover, *in cellulo* assays showed that both CVL218 and PJ34 treatments resulted in much more amount of puncta but no change in the occupied area (Figs. 2D, 2E and S7).

**Figure 2:**
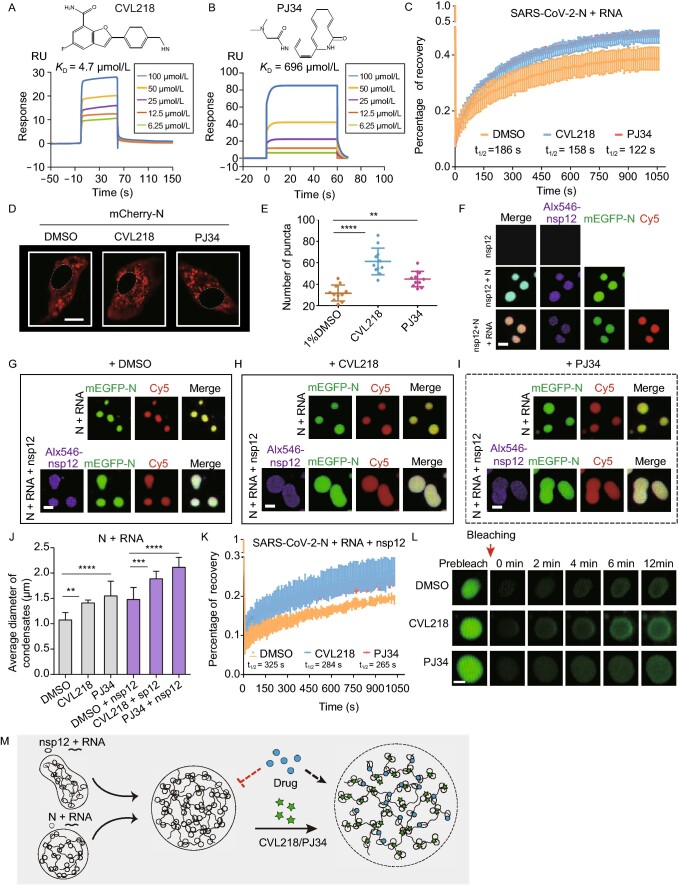
**CVL218 and PJ34 bind to SARS-CoV-2-N and influence the morphology and internal dynamics of the condensates of SARS-CoV-2 N-RNA-nsp12 complex**. (A and B) Surface plasmon resonance (SPR) assays of CVL218 (A) and PJ34 (B) to the immobilized full-length SARS-CoV-2-N. Top, the chemical structures of CVL218 and PJ34, respectively. Bottom, SPR binding curves of SARS-CoV-2 N to CVL218 and PJ34, respectively. (C) *In vitro* FRAP analysis of droplets (*n* = 3) formed by mEGFP-N protein with viral RNA (mEGFP-N, 3 μmol/L; RNA, 3 μmol/L) under the treatment of 20 μmol/L DMSO, CVL218 and PJ34, respectively. Data are representative of three independent experiments and presented as mean ± SD. (D) Droplet formation of the expressed mCherry-N (SARS-CoV-2 N tagged with mCherry) in Vero E6 cells after 48h transfection under three different treatments (20 μmol/L DMSO, CVL218 or PJ34, respectively). Scale bar, 10 μm. (E) Quantitative comparison of the numbers of droplets presented in (D). In total 11 transfected cells were considered in each treatment. Data are shown as mean ± SD and *P* values were determined by one-way analysis of variance (ANOVA) with Dunnett's multiple comparison test, **P < 0.01, ****P < 0.0001. (F) *In vitro* phase separation assays for 3 μmol/L Alex546 labeled nsp12 (purple) alone, with 3 μmol/L mEGFP-N (SARS-CoV-2 N tagged with mEGFP, green) and with the complex of 3 μmol/L mEGFP-N and 3 μmol/L Cy5-labeled viral RNA (red), respectively. The molar ratio between mEGFP-N and RNA was 1:1. Scale bar, 2 μm. (G–I) *In vitro* phase separation assays of 3 μmol/L mEGFP-N protein with 3 μmol/L viral RNA in the absence and presence of 3 μmol/L nsp12 under the treatment of 20 μmol/L DMSO (G), CVL218 (H) and PJ34 (I), respectively. Scale bar, 2 μm. (J) Quantification of the effect of CVL218 or PJ34 treatment on the average diameters of the condensates presented in (G–I). The diameters were measured from the fluorescence microscopy images and shown as mean±SD over three independent experiments. *P* values were determined by one-way analysis of variance (ANOVA) with Tukey's multiple comparison test, **P < 0.01, ***P < 0.001, ****P < 0.0001. (K) *In vitro* FRAP analysis of the condensates (*n* = 3) of SARS-CoV-2 N-RNA-nsp12 complex (mEGFP-N, 3 μmol/L; RNA, 3 μmol/L; nsp12, 3 μmol/L) under the treatment of 20 μmol/L DMSO, CVL218 and PJ34, respectively. Data are representative of three independent experiments and presented as mean ± SD. (L) Representative snapshots of the condensates before and after bleaching presented in (K). Scale bar, 2 μm. (M) A model mechanism of the inhibition of viral replication and transcription of SARS-CoV-2 by small molecules in a phase separation dependent manner. Nsp12 alone cannot undergo phase separation *in vitro*, but it can be recruited into the droplets of SARS-CoV-2 N-RNA complex, despite the fact that nsp12 and viral RNA can form solid-state condensates. Comparing to those with DMSO treatment, the diameters and mobility of SARS-CoV-2 N-RNA-nsp12 droplets obviously increased after the treatment of CVL218/PJ34, which can attenuate the local density of the condensates and thus promote the entrance of other antiviral drugs (e.g., remdesivir) into their targets (e.g., nsp12/RdRp). The reaction buffer of *in vitro* phase separation assays in (F–L) consists of 20 mmol/L HEPES, pH 7.4, 150 mmol/L NaCl, 5% glycerol, 1 mmol/L EGTA and 1 mmol/L MgCl_2_

So far, N protein is the only known structural protein of coronavirus that shuttles into and outside the replication and transcription complexes (RTCs) and plays a vital role in coordination with the RdRp complex (Cong et al., 2020). However, the underlying mechanisms remain largely obscure. To further explore whether N-driven LLPS is involved in these biological processes, we further purified nsp12, a core component of RNA dependent RNA polymerase (RdRp) complex. Under the physiological salt condition, nsp12 cannot undergo phase separation spontaneously (Fig. 2F), and readily converted to amorphous condensates with poor dynamic performance even mixed with viral RNA (Fig. S8A and Video S4) Consistently, the overexpressed GFP-nsp12 in Vero E6 cells aggregated into asymmetry condensates of dense structure in the cytoplasm (Fig. S8B). Nevertheless, we found that nsp12 can be readily recruited into the SARS-CoV-2-N-RNA condensates without changing their morphological shapes and arrangements (Fig. 2F). Similar results were also obtained using the RdRp complex, which was assembled *in vitro* by nsp12, nsp7 and nsp8 at a molar ratio of 1:1:2 (Fig. S9). Interestingly, the ubiquitin-like domain 1 (Ubl1) of nsp3 interacting with N protein (Hurst et al., 2013; Cong et al., 2020), was also verified to colocalize with the condensates of SARS-CoV-2 N-RNA complex (Fig. S10), implying that the N-driven LLPS might play an important role during the SARS-2-CoV life cycle.

Considering the effects of CVL218 or PJ34 on the N-driven LLPS, we examined their influence on the phase condensation properties of SARS-CoV-2 N-RNA-nsp12 complex. Interestingly, CVL218 or PJ34 treatment resulted in more increased sizes of SARS-CoV-2 N-RNA-nsp12 condensates than those of the DMSO-treated groups (Fig. 2H–J). More importantly, no matter with or without nsp12, the sizes of SARS-CoV-2 N-RNA condensates under CVL218 or PJ34 treatment were significantly larger than those of the DMSO treated group (Fig. 2G–J). Moreover, FRAP assays indicated that the fluorescence recovery rates of SARS-CoV-2 N-RNA-nsp12 condensates were faster in the CVL218 or PJ34 treated group than those of the control treatment (Fig. 2K and 2L). Meanwhile, *in cellulo* analysis revealed that CVL218 or PJ34 treatment significantly improved the colocalization of N protein with nsp12 (Fig. S11). Considering the much more complicated intracellular environment compared to the *in vitro* reaction system and the possible involvement of other host factors, it would be generally difficult to figure out the detailed interplay between SARS-CoV-2 N and nsp12 during the viral life cycle. Nevertheless, the effects of CVL218 or PJ34 treatment on the phase separation properties of SARS-CoV-2 N and the associations with nsp12 implied the possible roles of these two compounds as a selective partition to improve the pharmacodynamics of other anti-viral drugs (e.g., remdesivir, specifically targeting nsp12/RdRp).

Inspired by a recent study emphasizing the partition of antineoplastic drugs in specific protein condensates (Klein et al., 2020), we speculated that CVL218 and PJ34 may act as bulking agents to reduce the local density of the SARS-CoV-2 N-nsp12 condensates, and the increasing penetrability thus contributes to the access of other small-molecule drugs into the condensates (Fig. 2M).To explore this hypothesis, we evaluated the inhibitory activities of remdesivir combined with CVL218 against SARS-CoV-2 (Fig. S12) in Vero E6 cells. In particular, CVL218 and remdesivir were mixed with a concentration ratio of 4:1, while CVL218 or remdesivir alone was included as controls. We observed that the EC_50_ value of remdesivir measured from our experiment decreased from 1.41 μmol/L alone to 0.73 μmol/L in mixture, and the EC_50_ value of CVL218 decreased from 3.46 μmol/L alone to 2.93 μmol/L in mixture. This result indicated that the combination of CVL218 and remdesivir can enhance the therapeutic efficacy of individual drugs alone against SARS-CoV-2, which thus can partially support the model mechanism of antiviral activities against SARS-CoV-2 for the reported drugs in a phase separation manner (Fig. 2M).

In summary, our results indicated that the N protein-driven LLPS is a promising target for the design of antiviral drugs, and the deep understanding of the functional roles of N protein in regulating the accessibility of RTCs will thus advance the development of anti-SARS-CoV-2 therapies.

## Supplementary Information

The online version of this article (https://doi.org/10.1007/s13238-021-00832-z) contains supplementary material, which is available to authorized users.

## Supplementary Material

13238_2021_832_MOESM1_ESM Supplementary Materials

13238_2021_832_MOESM2_ESM Supplementary Table 1. Primers used in this study

13238_2021_832_MOESM3_ESM

13238_2021_832_MOESM4_ESM

13238_2021_832_MOESM5_ESM

13238_2021_832_MOESM6_ESM
