# Supplementary material for: Understanding the phase separation characteristics of nucleocapsid protein provides a new therapeutic opportunity against SARS-CoV-2
Source: Protein Cell. 2021 Mar 26;12(9):734–40. doi: 10.1007/s13238-021-00832-z (PMC7994959; doi:10.1007/s13238-021-00832-z)
Supplement: 13238_2021_832_MOESM1_ESM — Supplementary Materials [file 13238_2021_832_moesm1_esm.pdf]

# Supplementary Materials

## Understanding the phase separation characteristics of nucleocapsid protein provides a new therapeutic opportunity against SARS-CoV-2

Dan Zhao[1,10], Weifan Xu[2,10], Xiaofan Zhang[1,10], Xiaoting Wang[3], Yiyue Ge[4], Enming Yuan[1], Yuanpeng Xiong[5], Shenyang Wu[6], Shuya Li[1], Nian Wu[1], Tingzhong Tian[1], Xiaolong Feng[7], Hantao Shu[1], Peng Lang[1], Jingxin Li[4], Fengcai Zhu[4,8], Xiaokun Shen[9], Haitao Li[10], Pilong Li[2,*] and Jianyang Zeng[1,11,*]

[1]Institute for Interdisciplinary Information Sciences, Tsinghua University, Beijing, China.

[2]Beijing Advanced Innovation Center for Structural Biology, Beijing Frontier Research Center for Biological Structure, Tsinghua University-Peking University Joint Center for Life Sciences, School of Life Sciences, Tsinghua University, Beijing, China.

[3]Silexon AI Technology Co., Ltd., Nanjing, Jiangsu Province, China.

[4]NHC Key laboratory of Enteric Pathogenic Microbiology, Jiangsu Provincial Center for Diseases Control and Prevention, Nanjing, Jiangsu Province, 210009, China.

[5]Bioinformatics Division, BNRIST/Department of Computer Science and Technology, Tsinghua University, Beijing, China

[6]Protein Preparation and Identification Facility, Technology Center for Protein Science, Tsinghua University, Beijing, China.

[7]Institute of Pathology, Tongji Hospital, Tongji Medical College, Huazhong University of Science and Technology, Wuhan, Hubei Province, China.

[8]Center for Global Health, Nanjing Medical University, Nanjing, Jiangsu Province, 210009, China.

[9]Convalife (Shanghai) Co., Ltd., Shanghai, China.

[10]MOE Key Laboratory of Protein Sciences, Beijing Advanced Innovation Center for Structural Biology, Beijing Frontier Research Center for Biological Structure, Tsinghua-Peking Joint Center for Life Sciences, Department of Basic Medical Sciences, School of Medicine, Tsinghua University, Beijing 100084, China.

[11]MOE Key Laboratory of Bioinformatics, Tsinghua University, Beijing, China.

[10]These authors contributed equally: Dan Zhao, Weifan Xu, Xiaofan Zhang.

*E-mail: pilongli@mail.tsinghua.edu.cn; zengjy321@tsinghua.edu.cn.

## Materials and Methods

### Cell culture and transfection

Vero E6 cells were kindly provided by Dr. Yiyue Ge and Dr. Jingxin Li from NHC Key Laboratory of Enteric Pathogenic Microbiology, Jiangsu Provincial Center for Disease Control and Prevention. Vero E6 cells were cultured in Dulbecco's modified Eagle's medium (HyClone) supplemented with 10% fetal bovine serum (HyClone SH30071.03 and SH30396.03) and maintained at 37°C in a humidified incubator with 5% $CO_2$. FuGENE 6 (Promega, E2691) was used for transient transfection according to the manufacturer's instructions. The SARS-CoV-2 virus strain used in this study was BetaCoV/JS03/human/2020 (EPI ISL 411953), which was isolated from a 40-year old female confirmed as a COVID-19 case in December 2019. Vero E6 cells were used to propagate the virus, and the viral titer was measured by the 50% tissue culture infective dose (TCID50) through microscopic observation of cytopathic effect. All the SARS-CoV-2 infection related experiments were performed in a biosafety level-3 (BLS-3) laboratory in Jiangsu Provincial Center for Diseases Control and Prevention, Jiangsu, China.

### Construction of recombinant plasmids

The recombinant plasmids of pET28a-N and pET22b-nsp12 were kindly provided by Cellregen Co., Ltd. and Prof. Zhiyong Lou (Gao et al., 2020), respectively. The mutants of pET28a-N were constructed by seamless cloning kits (Beyotime, D7010M) and confirmed by sequencing. For the construction of mEGFP-N plasmids, the full-length gene and truncations of SARS-CoV-2 N were both cloned into a PL118 vector (an in-house modified vector based on pRSFDuet1) containing an N-terminal 6×his-mEGFP tag, respectively. The full-length gene of SARS-CoV-2 N was cloned into a pCDNA3.1 vector containing an N-terminal mCherry tag. The full-length gene of SARS-CoV-2-Nsp12 was cloned into a pCDNA3.1 vector containing an N-terminal GFP tag. The Ubl1 domain of SARS-CoV-2 nsp3 (1-112 aa) was

cloned into a pGEX-4T-2 vector. The detailed primer sequences are listed in Supplementary Table S1.

**Protein expression and purification**

The recombinant full-length mEGFP-N protein and truncations were overexpressed in *E. coli* BL21 (DE3). After overnight induction by 0.2mM isopropyl β-D-thiogalactoside (IPTG) at 16 °C in LB medium, cells were harvested and suspended in the buffer (40mM HEPES, pH 7.5, 1M NaCl, 20mM imidazole and 2mM phenylmethylsulfonyl fluoride). After cell lysis and centrifugation, the recombined proteins were purified to homogeneity over HisTrap column and eluted with a linear imidazole gradient from 20 mM to 500 mM. The proteins were further purified by size-exclusion chromatography using a Superdex 200 Increase 10/300 GL column (GE Healthcare) in elution buffer (40 mM HEPES, pH7.5, 1M NaCl, 5% glycerol, 1 mM EGTA, 1 mM $MgCl_2$). The purification procedures of the recombinant wild-type pET28a-N protein and mutants were essentially the same as that of the mEGFP-N protein except for a different size-exclusion chromatography buffer (20mM Tris, pH 7.5, 300mM NaCl).

The Ubl1 domain of nsp3 gene (1-112 aa) of SARS-CoV-2 was cloned into a modified pGEX-4T-2 vector, with a GST tag. The plasmids were transformed into *E. coli* BL21 (DE3). After overnight induction by 0.2mM IPTG at 16 °C in LB medium, the cells were harvested and resuspended in lysis buffer (20 mM Tris-HCl, pH 7.5, 500 mM NaCl, 20mM Imidazole) and homogenized with an Emulsiflex C3 (Avestin) high-pressure homogenizer at 4 °C. After centrifugation at 13,000 rpm, the supernatant was loaded on a GST column and the fusion protein was directly digested on the column overnight by the Thrombin protease (Beijing Solarbio Science & Technology, T8021). The resultant protein was further loaded on a Superdex 75 10/300 Increase column (GE Healthcare, USA) in a buffer containing 40 mM HEPES,

pH 7.5, 1mM EGTA, 150 mM NaCl, 1 mM $MgCl_2$ and 5% glycerol. The purified Ubl1 domain of nsp3 was concentrated and stored at -80 °C.

**Assembly of RdRp complex *in vitro***

The purification and assembly of RdRp complex of SARS-CoV-2 were essentially the same as described previously (Gao et al., 2020) with minor modifications. Briefly, the nsp12 gene was cloned into a modified pET22a vector, with the C-terminus possessing a 10 × His-tag. The plasmids were transformed into *E. coli* BL21 (DE3), and after overnight induction by 0.2mM IPTG at 16 °C in LB medium, cells were harvested and suspended in the buffer (20 mM Tris-HCl, pH 7.5, 500 mM NaCl, 20mM Imidazole) and homogenized with an Emulsiflex C3 (Avestin) high-pressure homogenizer at 4 °C. After centrifugation at 13,000 rpm, the supernatant was loaded on Ni-NTA column (GE Healthcare, USA) and then further purified by a Hitrap Q ion-exchange column (GE Healthcare, USA). The peaks eluted were loaded on a Superdex 200 10/300 Increase column (GE Healthcare, USA) in a buffer containing 40 mM HEPES pH 7.5, 1mM EGTA, 150 mM NaCl, 1 mM $MgCl_2$ and 5% glycerol. The purified nsp12 was concentrated and stored at 4 °C. Full-length nsp7 (without purification tag) and nsp8 (with a 6 × His-SUMO tag) of SARS-CoV-2 were co-expressed in *E. coli* BL21 (DE3) cells. After purification by Ni-NTA affinity chromatography, the nsp7-nsp8 complex was eluted through on-column tag cleavage by ULP protease and further loaded on a Superdex 200 10/300 Increase column (GE Healthcare, USA) in a buffer containing 40 mM HEPES pH 7.5, 1mM EGTA,150 mM NaCl, 1 mM $MgCl_2$ and 5% glycerol.

For the assembly of RdRp, purified nsp12 was incubated with nsp7 and nsp8 at 4 ℃ for 3 hours at a molar ratio of 1:2:2 in a buffer containing 40 mM HEPES pH 7.5, 1mM EGTA, 150 mM NaCl, 1 mM $MgCl_2$ and 5% glycerol. The stable complex sample was further purified by a Superdex 200 10/300 Increase column (GE

Healthcare, USA) in a buffer containing 40 mM HEPES pH 7.5, 1mM EGTA ,150 mM NaCl, 1 mM $MgCl_2$ and 5% glycerol.

**Protein labeling**

All pET28a-N proteins (WT and mutants), nsp12, RdRp complex (nsp12-nsp7-nsp8) and nsp3-Ubl1 proteins were labeled by incubating with a 1:1 molar ratio of Alexa Fluor™ 546 carbox (Thermo Fisher Scientific) for 1 h at room temperature with continuous stirring. Then, the free dyes were removed by centrifugation in MICROSPIN G-50 column (GE Healthcare, 27-5330-01). The labeled proteins were stored at -80°C. For *in vitro* phase separation experiments, 5% labeled protein was mixed with unlabeled before use.

**Synthesis of RNA and DNA**

The 5’-Cy5-labeled 30-bp RNA oligos (viral RNA: GAUUUCAUCUAAACGAACAAACUAAAAUGU; human β actin RNA: UCACCAACUGGGACGACAUGGAGAAAAUCU) were synthesized at HIPPOBIO, LLC. The double-strand RNA was annealed at 25 µM in the annealing buffer (40 mM HEPES, pH 7.4, and 150 mM NaCl) using a thermocycler, during which the oligos were heated up to 95 °C for 2 min and gradually cooled to 25°C over an hour.

**Phase separation assays**

*In vitro* LLPS experiments were performed at room temperature. All samples were seeded and recorded on 384 low-binding multi-well 0.17 mm microscopy plates (In Vitro Scientific) and sealed with optically clear adhesive film. For phase separation assays with the mEGFP-N protein of SARS-CoV-2, solutions of GFP fusion proteins were diluted to the indicated final concentrations in 20 mM HEPES, pH 7.4, 150 mM NaCl, 5% glycerol, 1 mM EGTA and 1mM $MgCl_2$ in a total volume of 10 µl to

induce phase separation. For the N proteins without tags, the assays were performed in 20 mM Tris-HCl, pH 7.5 and 150 mM NaCl. For phase separation assays treated with small molecules, CVL218 or PJ34 (dissolved in 1% DMSO) were added to the well mixed phase separation samples prior to imaging at a final concentration of 20 μM. The group treated with 1% DMSO was used as the control.

For *in cellulo* assays, Vero E6 cells were seeded into 4-well chamber 35 mm dishes with a density of $5 \times 10^5$ cells/well. For cells to reach 70% confluent, 1 μg pCDNA3.1-mcherry-N plasmid was transfected, with the replacement of normal cell culture medium by that supplemented with CVL218 or PJ34 at a final concentration of 20 μM. For the control wells, cell medium containing 1% DMSO was added. Imaging was performed with a NIKON A1 microscope equipped with a 100× oil immersion objective. NIS-Elements AR Analysis was used to analyze the images.

**Fluorescence recovery after photobleaching (FRAP) measurements *in cellulo* and *in vitro***

FRAP experiments were carried out with a NIKON A1 microscope equipped with a 100× oil immersion objective. Droplets were bleached with the corresponding laser pulse (3 repeats, 80% intensity, and dwell time 1 s). Recovery from photobleaching was recorded for the indicated time point.

**Mutation frequency analysis**

To perform the mutation frequency analysis of SARS-CoV-2 N protein, we used 61,003 SARS-CoV-2 genome sequences downloaded from the China National Center for Bioinformation, 2019 Novel Coronavirus Resource(Zhao et al., 2020) (downloaded on July 6th, 2020). We considered all the missense mutations among the N protein region (from positions 28,274 to 29,530 in the genome).

**Surface plasmon resonance assays**

Surface plasmon resonance (SPR) assays were performed on Biacore S200 with a CM5 sensor chip (GE Healthcare Life Sciences) at room temperature. The full-length N protein, NTD and CTD of SARS-CoV-2 were all diluted in 10 mM sodium acetate (pH 5.0) and immobilized on a CM5 sensor chip by amine coupling. The running buffer contained 1×PBS-P with 2% DMSO. The tested drugs (CVL218 or PJ34) in 2-fold serial dilutions were made in the running buffer. The solutions flowed through the chip surface at a flow rate of 20 μL/min at room temperature (25°C). The dissociation constants ($K_D$) of CVL218 and PJ34 binding to full-length N protein, NTD and CTD were calculated from the association and dissociation curves of the sensorgrams using the BIA evaluation program (Biacore).

**Inhibitor combination assay**

To assess the potential synergistic effect, CVL218 and remdesivir were mixed with a concentration ratio of 4:1, while CVL218 or remdesivir alone was used as control. The concentration ratios were selected according to the corresponding EC25 values of individual drugs against SARS-CoV-2 *in vitro*. The mixtures were tested for their inhibitory activities against the SARS-CoV-2 with a multiplicity of infection (MOI) of 0.05. Each sample was tested in triplicate.

## Supplementary Figures

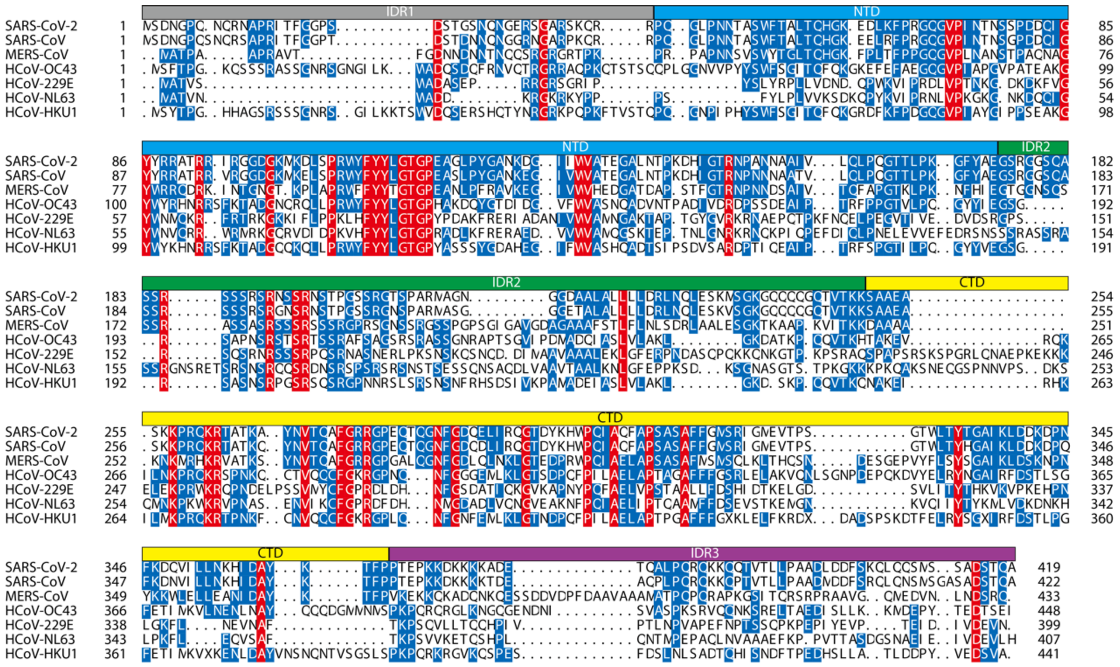

**Figure S1. Multiple sequence alignment of the N proteins from different human coronaviruses.**

The nucleocapsid (N) protein sequences of the currently known coronaviruses that can infect human, including SARS-CoV-2 (GenBank: QHD43423.2), SARS-CoV (GenBank: AYV99827.1), MERS-CoV (GenBank: AVV62544.1), HCoV-OC43 (GenBank: AAR01019.1), HCoV-229E (GenBank: APD51511.1), HCoV-NL63 (NCBI Reference Sequence: YP_009328939.1) and HCoV-HKU1 (GenBank: ARU07581.1), were aligned using MUSCLE(*38*). Domain architectures are depicted above the sequence alignment. The conserved residues are shaded in red, while those with the percentage of conservation larger than or equal to 50% are shaded in blue.

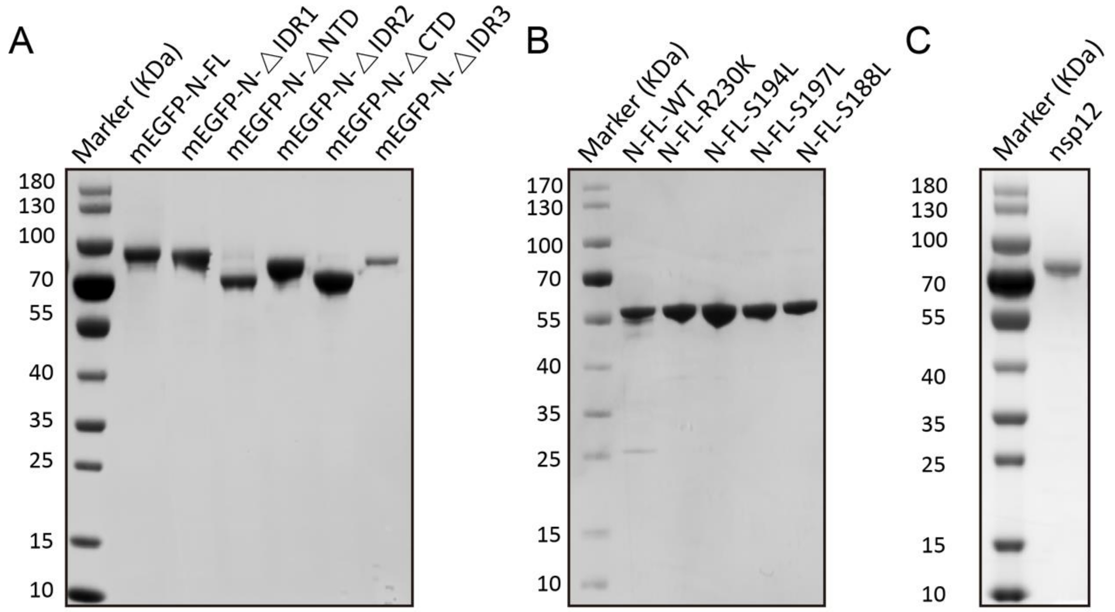


**Figure S2. SDS-PAGE of the purified recombinant proteins of SARS-CoV-2 N and nsp12 used in *in vitro* assays.**

**(A)** The mEGFP-tagged full-length (FL) and truncated proteins of SARS-CoV-2 N. **(B)** The wild-type and mutant proteins of SARS-CoV-2 N with His-tagged at the N terminus. **(C)** The nsp12 protein of SARS-CoV-2. The gel was stained with Coomassie Brilliant Blue.

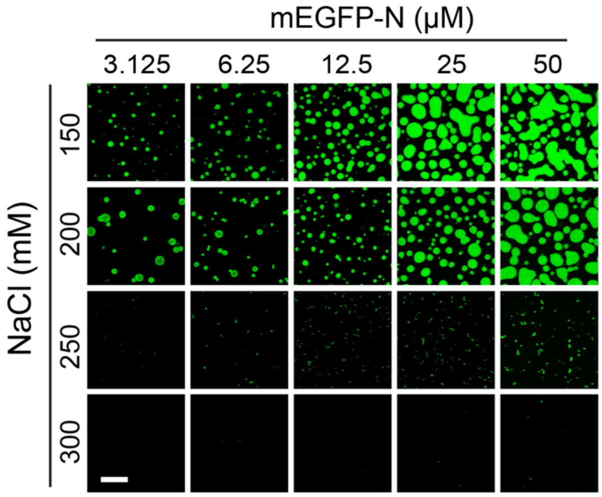


**Figure S3. Phase condensation of SARS-CoV-2 N was sensitive to the increase of ionic strength.**

Fluorescence microscopy observations of mEGFP-N condensates *in vitro* depending on different sodium chloride concentrations and protein concentrations. Scale bar, 20 µm.

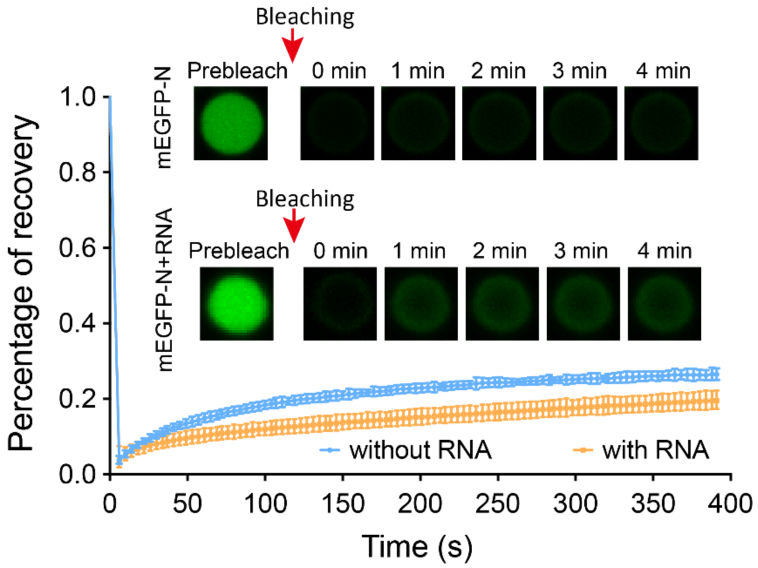


**Figure S4. *In vitro* FRAP analysis of mEGFP-N condensates (3 µM) with a complete bleach region.**

Top, representative snapshots of condensates before and after bleaching. Bottom, average fluorescence recovery traces of mEGFP-N condensates. Data are representative of three independent experiments and presented as mean±SD.

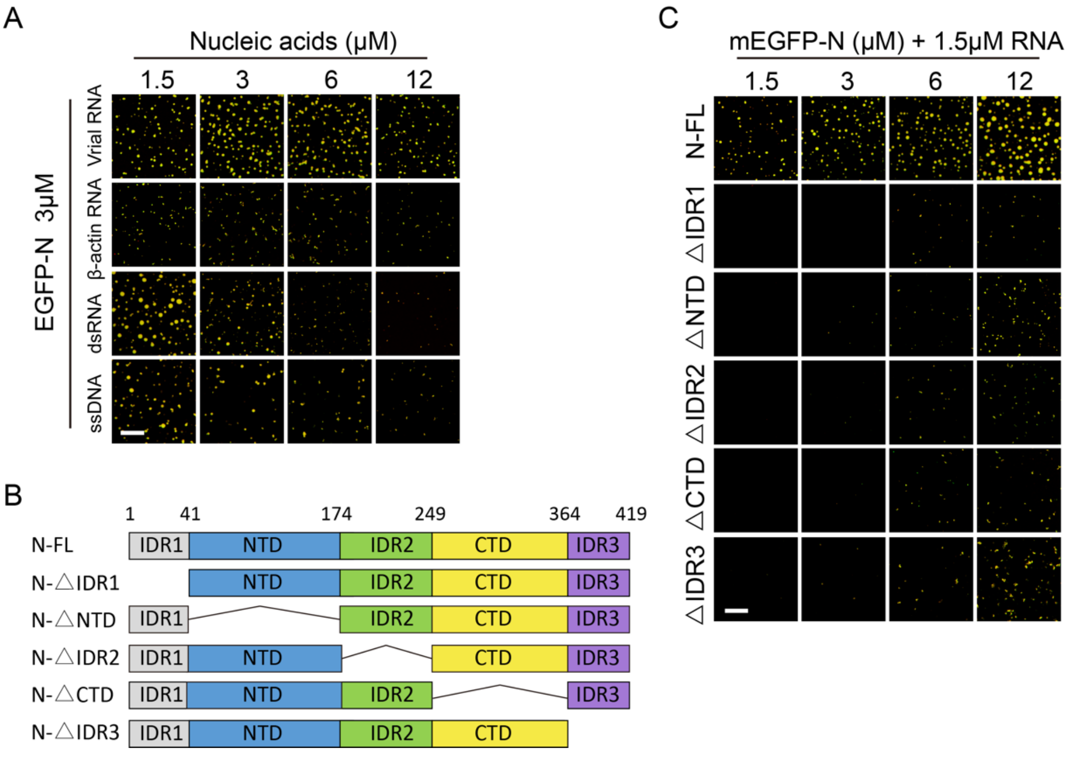


**Figure S5. Phase diagrams of RNA with SARS-CoV-2 N and truncations.**

(A) *In vitro* phase separation assays of mEGFP-N with nucleic acids from distinct sources and at different concentrations. Only the merged channel is shown here. Scale bar, 20 µm. (B) Diagram of the structural domains of SARS-CoV-2 N. Truncated proteins for functional analyses of different domains are shown underneath. (C) *In vitro* phase separation assays of full-length (FL) mEGFP-N and truncations with 1.5 µM viral RNA at different concentrations of SARS-CoV-2 N (The numbers under the line represent the concentrations of the proteins). Scale bar, 20 µm.

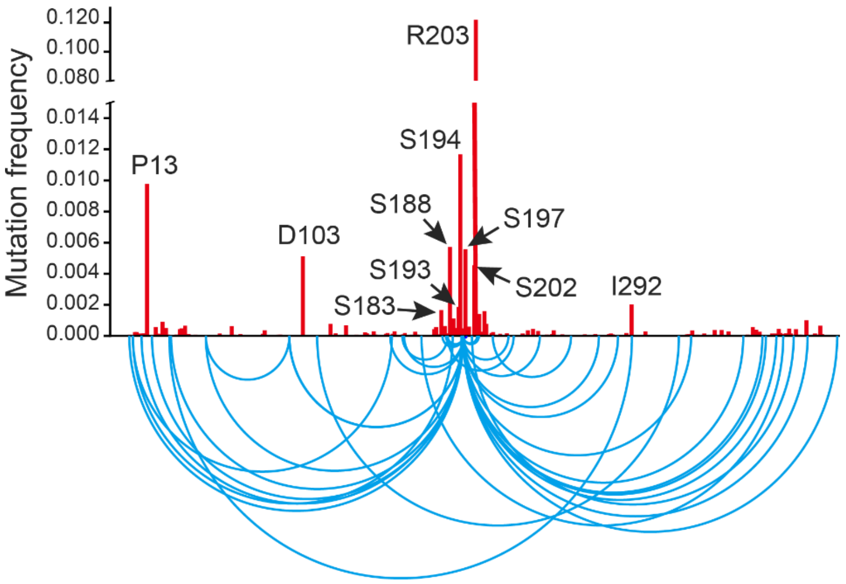


**Figure S6. Frequencies of spontaneous missense mutations in the N protein in 61,003 SARS-CoV-2 genome sequences from the China National Center for Bioinformation.**

Residue positions of the top 10 most frequent missense mutations are labeled. Bottom, arc diagram of double missense mutations. Only those double missense mutations with frequencies more than 0.0001 are shown.

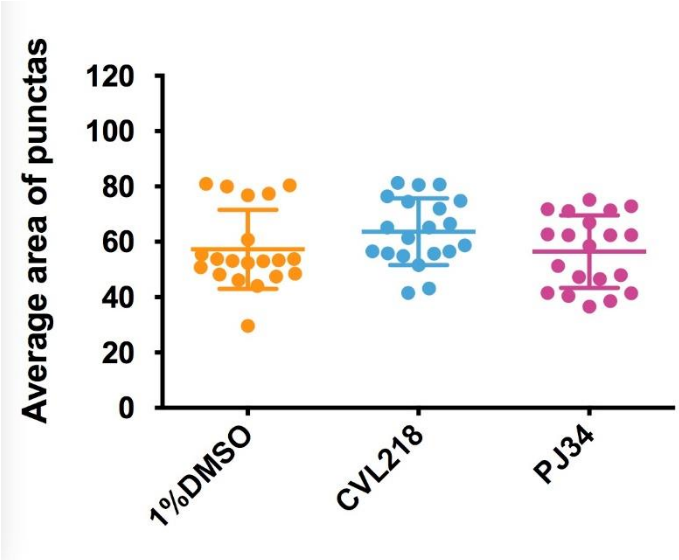


**Figure S7. Quantitative comparison of the average area of N protein puncta *in cellulo*.**

Total 20 puncta within five Vero E6 cells transfected with mCherry-N were measured in each treatment to compare the average area of puncta. Data are shown as mean ± SD using ImageJ analysis.

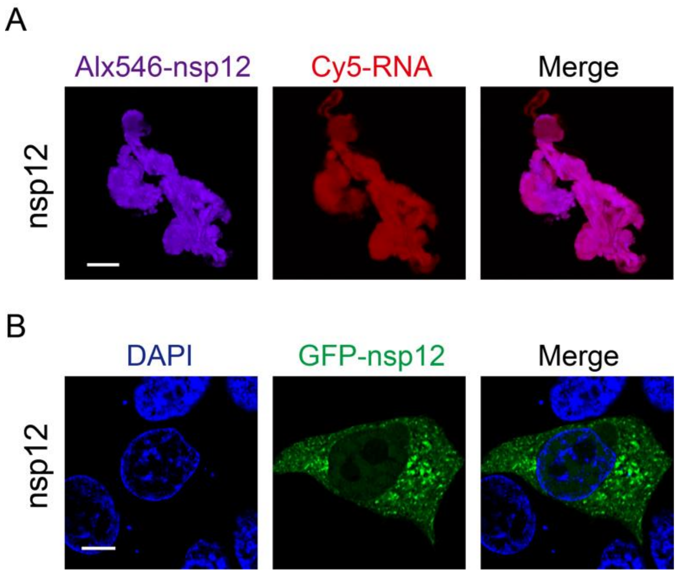


**Figure S8. Nsp12 and viral RNA of SARS-CoV-2 form amorphous condensates *in vitro*.**

**(A)** Fluorescence microscopy images of 3 μM Alx546-labeled nsp12 (purple) mixed with 3 μM Cy5-labeled viral RNA (red) of SARS-CoV-2. Scale bar, 5 μm. **(B)** Locations of overexpressed GFP-nsp12 in Vero E6 cells after 48h transfection. The nuclei were labeled by DAPI. Scale bars, 5 μm.

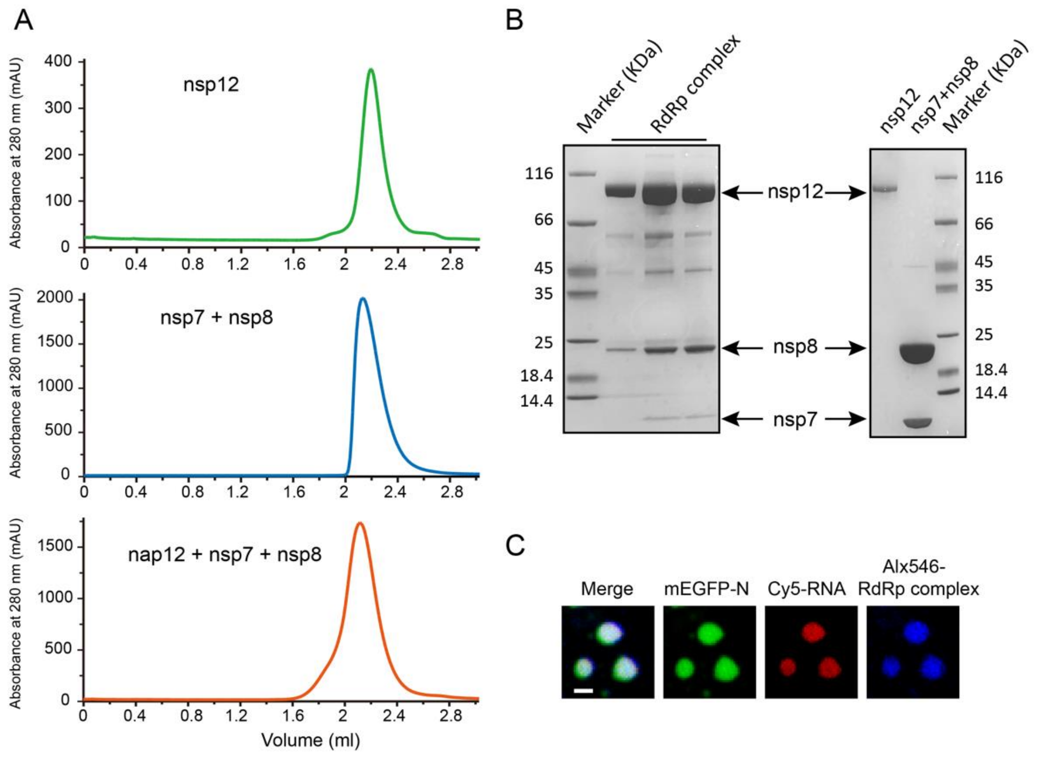


**Figure S9. Purification of SARS-CoV-2 polymerase catalytic complex and its phase separation with the nucleocapsid protein.**

**(A)** Gel filtration chromatography analysis of nsp12, nsp7-nsp8 complex and assembled RdRp complex (nsp12+nsp7+nsp8) by Superdex 200 Increase 5/150 GL column. **(B)** SDS-PAGE analysis of the chromatography peaks according to **(A)**. **(C)** *In vitro* phase separation assay for 3 μM mEGFP-N protein with 3 μM Cy5-labeled 30-nt viral RNA in the presence of 3 μM RdRp complex. Scale bar, 2 μm.

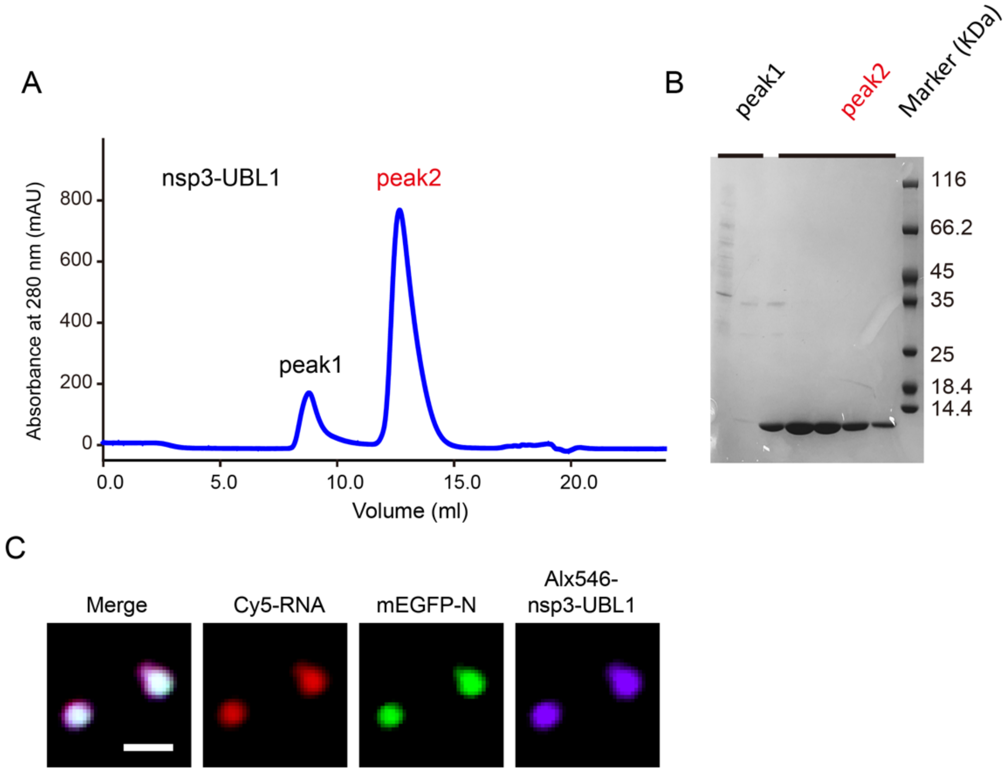


**Figure S10. The Ubl1 domain of SARS-CoV-2 nsp3 can be recruited by nucleocapsid protein *in vitro*.**

**(A)** Gel filtration chromatograph analysis of nsp3-Ubl1 by Superdex 75 Increase 100/300 GL column. **(B)** SDS-PAGE analysis of the chromatograph peaks corresponding to **(A)**. **(C)** *In vitro* phase separation assay of 3 μM mEGFP-N protein with 3 μM Cy5-labeled 30-nt viral RNA in the presence of 3 μM nsp3-Ubl1 protein. Scale bar, 2.5 μm.

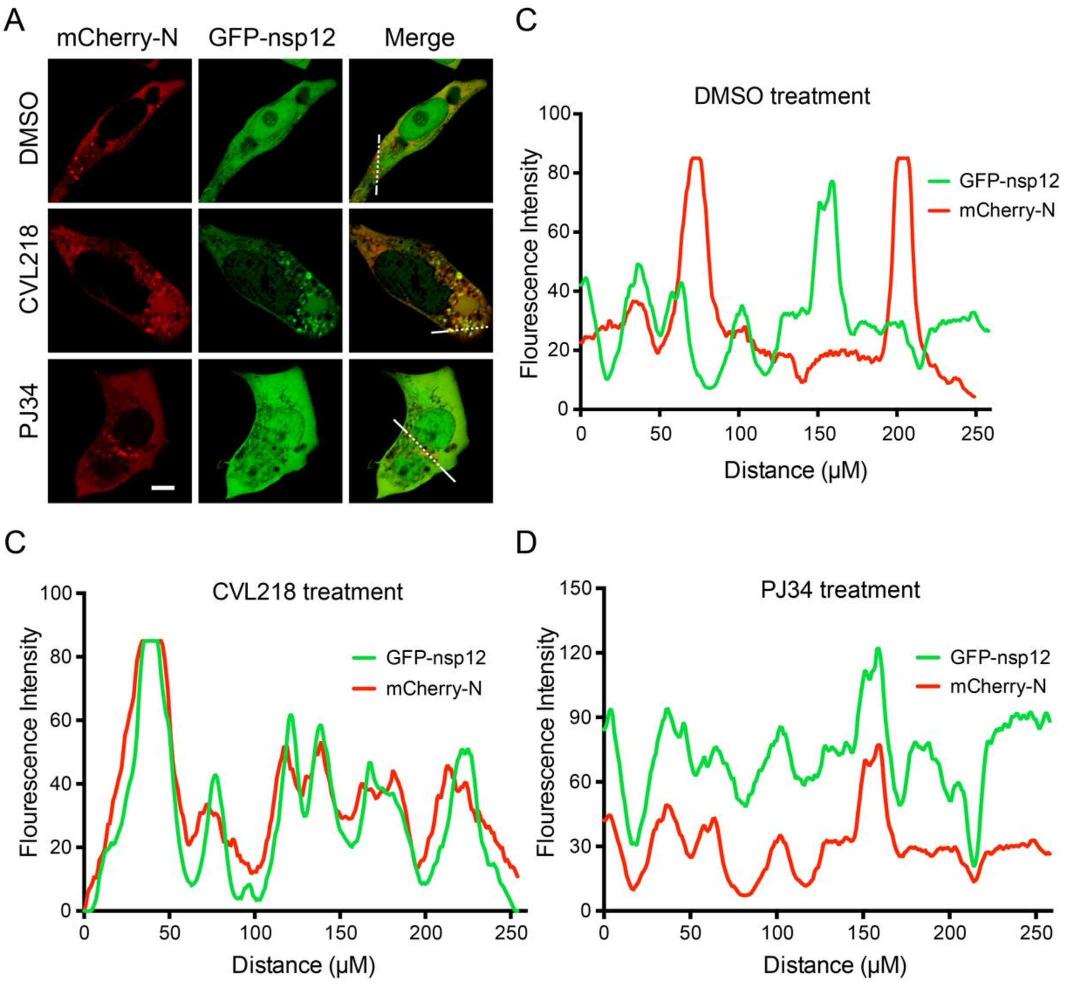


**Figure S11. CVL218/PJ34 treatment enhances the co-localization of SARS-CoV-2 N and nsp12 in Vero E6 cells.**

**(A)** Locations of overexpressed GFP-nsp12 (green) and mCherry-N (red) in Vero E6 cells after 48h transfection under three different treatments (DMSO, CVL218 or PJ34, 20 μM, respectively, 20um). The nucleus was labeled by DAPI. Scale bar, 5 μm. **(B-D)** Co-localization analyses of SARS-CoV-2 N and nsp12 in cells under three different treatments (DMSO, CVL218 or PJ34, respectirely). The fluorescence intensities were measured along the dotted lines in the merged images shown in **(A)**.

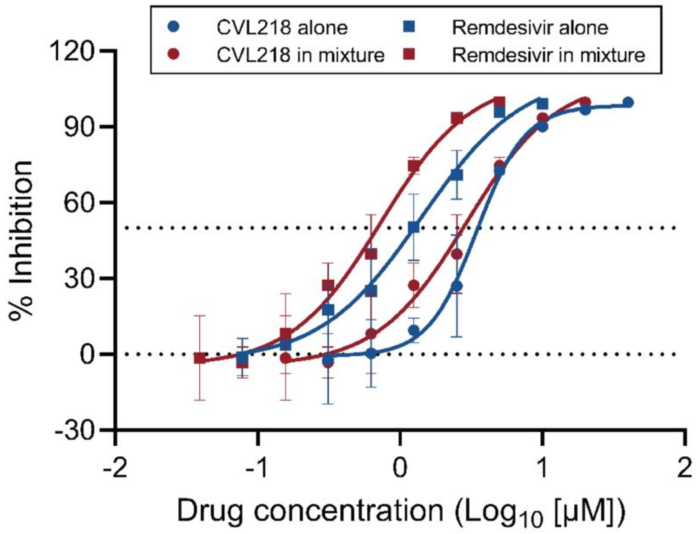


**Figure S12. The antiviral activities of the tested drugs against SARS-CoV-2 in Vero E6 cells.**

Vero E6 cells were infected with SARS-CoV-2 at an MOI of 0.05 in the treatment of different doses of the indicated drugs for 48h. The viral yield in cell supernatant was quantified by qRT-PCR and the results are shown as mean ± SD over three independent experiments. For the drug combination assays, CVL218 and remdesivir were mixed with a concentration ratio of 4:1. The EC50 values inhibiting the replication of SARS-CoV-2 for remdesivir alone, remdesivir in mixture, CVL218 alone and CVL218 in mixture are 1.41 μM, 0.73 μM, 3.46 μM and 2.93 μM, respectively.
